# Phosphoric Acid in Organic Nature

**Published:** 1869-11

**Authors:** 


PHOSPHORIC ACID IN ORGANIC NATURE.—In a recent number of the *Scientific American* it is remarked that the exudates of plants generally contain no phosphoric acid; at least such is the case with manna and gum-arabic. It is known that in exhausting the pulp of young roots with water, fibrin is obtained, which contains pectose and the incrusting substances. It follows, therefore, that the skeleton of vegetables owes its solidity not to the phosphates, as is the case with that of animals. The leaves that remain in the forests during winter yield ashes rich in iron, silica and lime, but free of phosphorus. It is also worthy of note that, although analysis has as yet failed to discover phosphates in the sea, the maritime plants contain considerable quantities of this substance.

Corenwinder, at least, has searched in vain for phosphoric acid in the water of the North Sea, as well as in the boiler sediments of vessels crossing the ocean. The pollards of flowers and the spores of cryptogams are rich in the acid of phosphorus; this being especially the case with the pollards of *Lilium candium*. It is remarkable that the ashes of pollards and those of the semen of animals are nearly allied in their component parts, they being both rich in phosphoric acid.

